# An Ultra-Low Power and Flexible Acoustic Modem Design to Develop Energy-Efficient Underwater Sensor Networks

**DOI:** 10.3390/s120606837

**Published:** 2012-05-25

**Authors:** Antonio Sánchez, Sara Blanc, Pedro Yuste, Angel Perles, Juan José Serrano

**Affiliations:** Instituto ITACA, Universitat Politècnica de València, Camino de Vera s/n 46022, Valencia, Spain; E-Mails: ansanma7@itaca.upv.es (A.S.); pyuste@disca.upv.es (P.Y.); aperles@disca.upv.es (A.P.); jserrano@itaca.upv.es (J.J.S.)

**Keywords:** underwater sensor networks, wireless sensor networks, acoustic modems, wake-up, underwater MAC

## Abstract

This paper is focused on the description of the physical layer of a new acoustic modem called ITACA. The modem architecture includes as a major novelty an ultra-low power asynchronous wake-up system implementation for underwater acoustic transmission that is based on a low-cost off-the-shelf RFID peripheral integrated circuit. This feature enables a reduced power dissipation of 10 μW in stand-by mode and registers very low power values during reception and transmission. The modem also incorporates clear channel assessment (CCA) to support CSMA-based medium access control (MAC) layer protocols. The design is part of a compact platform for a long-life short/medium range underwater wireless sensor network.

## 1. Introduction

There is an increasing need for short-range, long-life, low-cost underwater communications systems that provide flexibility and maintenance-free installations. Typical applications for these requirements are water pollution monitors, offshore fish farms, autonomous underwater vehicle guidance, water parameter data-logging, and coastal surveillance applications, *etc.* These types of applications may require one or more of the following: short range underwater communications between collaborative devices; short/medium range communications between sea bottom and surface; and longer range communications between the site and land. In these applications it is often desirable to eliminate or reduce the number of wires and connectors to a minimum to reduce cost and maintenance and increase reliability.

There are important research projects on wireless sensor networks in marine environments such as [1–3], or for relevant ecosystems [4,5], and these point to the need for specific systems for environmental monitoring of water parameters in lakes, bays, ports, seas, and oceans.

Practical solutions mainly use floating buoys with sensors attached to data bus cables for vertical measure profiling; and buoy wireless communication is achieved aerially using ISM frequency bands as in [6–10]. Although surface buoys are interesting, the development of underwater wireless sensor networks (UWSN) requires more flexible generic solutions using submerged or floating modems that can be adapted to many scenarios [11].

Acoustic communication is the current choice for distances over one meter when deploying a submerged sensor that communicates wirelessly. This approach offers greater reliability and range than radio frequency or optical alternatives [12]. An acoustic modem sensor, in the context given above, must be self-powered because replacement is difficult once deployed, and so an energy-efficient design is required to extend its life for months or even years.

Current difficulties in finding an adequate underwater acoustic modem prompted us to design a flexible, ultra-low power, low-cost modem whose architecture is focused on long-life submerged sensoring nodes and which supports energy efficient communication protocols. The modem, called ITACA [13], provides transmission of digital data using coherent-FSK at rates of 1 kbps with an 85 kHz carrier frequency. The modem only requires 11 μW on stand-by and 24 mW in data reception mode—and this is currently the lowest reported power requirement. Range depends on transmission power. The objective is to reach short/medium distances with the lowest recorded power consumption, and in October 2011 a distance of 240 meters was experimentally achieved inside a small marina with a transmission power of 108 mW.

The proposal presented in this paper is focused on designing an aquatic physical platform in an energy-efficient way that enables the efficient development of upper communication layers by supporting additional RTC (Real Time Clock) asynchronous wake-up and clear channel assessment (CCA) detection using both carrier frequency detection and the RSSI level.

This paper is organized as follows. State-of-the-art related work is described in Section 2. Section 3 describes the acoustic modem architecture block-by-block. Section 4 discusses energy analysis while Section 5 concludes the paper.

## 2. Related Work

Although most terrestrial wireless sensor networks are based on electromagnetic wave propagation, the extrapolation of this technology to an underwater environment as shown in [14] is not promising due to the huge electromagnetic wave attenuation and, consequently, the high power level required to achieve acceptable transmission ranges between modems. A more interesting option in terms of price, power consumption, and network reliability is the use of acoustic modems.

Some approaches use common microphones and speakers to perform the conversion of electric signals into acoustic signals, and *vice versa* [15,16]. However, the efficiency of these transducers is quite low and an alternative should be explored to produce the conversion. The transduction from electrical energy to mechanical energy can be efficiently accomplished by piezoelectric materials such as those found in relatively cheap piezoelectric based echo-sounder devices.

Some alternatives have been explored to properly excite piezoelectric materials for effective acoustic communication [17]. However, an ultra-low consumption solution is still needed for a modem architecture to obtain a balance between cost, consumption, and medium range distance rates for both peer-to-peer connections and dense networks.

Communication over long link distances has already been achieved with modems, including commercial models such as the Tritech (up to 8 km) [18], the WHOI modem (1–10 km) [19], or UCSD (greater than 1 km) [20]. These modems can also be used for short or medium distances. However, their consumption is too high for deployment as reliable autonomous sensor networks using power supplied by small batteries for periods of months.

The lowest consumption is achieved in [21] and [22]. The development of energy efficient enhanced modems should take into consideration the physical layer and the medium access control (MAC) layer from the very early design stages. A modem whose physical layer consumes little, but which must support a complex or non-energy efficient MAC protocol loses all the benefits gained. For example, stand-by power consumption in modern microcontrollers can be very low. In more detail, ‘sleep mode’ reduces power consumption to a minimum by turning off the central processing unit (CPU) and attached peripherals. Therefore, exploring ‘sleep mode’ is an interesting approach for saving energy in underwater MAC policies. However, as messages cannot be sent or received by the wireless interface in sleep mode, the microcontroller needs to wake up and resume normal activity.

In this line of work, designs [21] and [22] present types of wake-up systems to maintain the microcontroller in sleep mode as often as possible. Strategies to wake-up (WU) a modem can be grouped into synchronous or asynchronous strategies. Synchronous WU strategies are based on time-sharing and synchronization, while asynchronous WU strategies depend on external stimuli.

Synchronous WU strategies need very simple additional hardware. Time synchronization can be achieved by using a real-time clock or even a timer. This hardware is widely available and usually dissipates very little power—and is even integrated as an on-chip peripheral in several state-of-the-art microcontrollers. Synchronous WU strategies are based on the premise that two modems that want to communicate will switch on their wireless interfaces at the same time by keeping their corresponding internal clocks synchronized. WU time-driven protocols achieve energy efficiency through the accuracy of their clock synchronization. In event-driven communication, which starts when some specific event is recognized, a wireless interface tends to remain active more time than strictly needed to complete packet transmission. Generally speaking, synchronous WU strategies consume more energy than the minimum required to transmit or receive. For example, some researchers have compared the STEM synchronous WU strategy [23] with an asynchronous wake-up strategy [24].

Asynchronous wake-up modem consumption has been compared with the utopian lowest possible consumption level in [24] and [25] and the conclusions are that asynchronous WU systems are optimal for reaching this low consumption level.

However, asynchronous WU strategies need specific hardware. A modem must be able to react to certain stimuli. For example, an acoustic asynchronous triggered wake-up (AAW-U) circuit is needed for an acoustic signal to reactivate the modem. This circuit must dissipate as little power as possible since it remains always active. Asynchronous wake-ups are close to the optimal case of opening the wireless interface only for packet transmission or reception in both time-driven and also event-driven protocols, and this represents an advantage over synchronous wake-up strategies that are mainly energy efficient with time-driven protocols. Within asynchronous wake-up strategies, when a modem wants to transmit a packet before sending a payload, the transmitter must wake the receiver or receivers by emitting a WU signal. Thus, a receiver can remain inactive, or asleep, and become active only when a WU signal is detected. Moreover, a receiver can also transmit in a multi-hop network as it is possible to coordinate reception with sensing and transmission, or coexist with synchronous programmed wake ups. In any case, a flexible physical platform where different communication polices can be configured depending on the application requirement is an important advance in UWSN deployment, especially if its architecture is coherent with limited power constraints.

However, very little work has been done on asynchronous AAW-U. The main reference found was in 2006 [21], although this wake-up circuit draws 500 μW when waiting for a valid incoming wake up signal. Currently, there are terrestrial WU circuits that dissipate less than 100 μW in stand-by mode. State-of-the-art asynchronous wake-up systems for WSN dissipate 52 μW [26], 11 μW [27] and 12.6 μW [28], although the final energy saved depends on range the work presented in [25] shows a theoretical analysis of RFID for underwater WU. The study shows that modem power consumption is close to an optimal consumption boundary when the RFID asynchronous WU is used. In the present paper, the authors go one step forward by implementing an AAW-U circuit based on RFID technology and testing it in a seawater environment.

Finally, clear channel assessment (CCA) is an additional capability for incorporation into a physical modem design. CCA is a physical layer mechanism that is essential to CSMA/CA MAC protocols. The advantages of using CCA to support medium access control algorithms have been reviewed in [29]. Before transmitting, a modem will listen to the channel to set it as either free or occupied [30]. The solution described in the paper takes advantage of both carrier frequency detection and the received signal strength indicator (RSSI) to detect energy in the communication channel. The solution is power-consumption aware because it does not require additional hardware and is implemented within the AAW-U module.

The overall architecture presented below is designed under consumption constraints and is intended to be a flexible platform for the development of underwater wireless sensor networks.

## 3. ITACA Acoustic Modem Architecture

**Figure 1.**
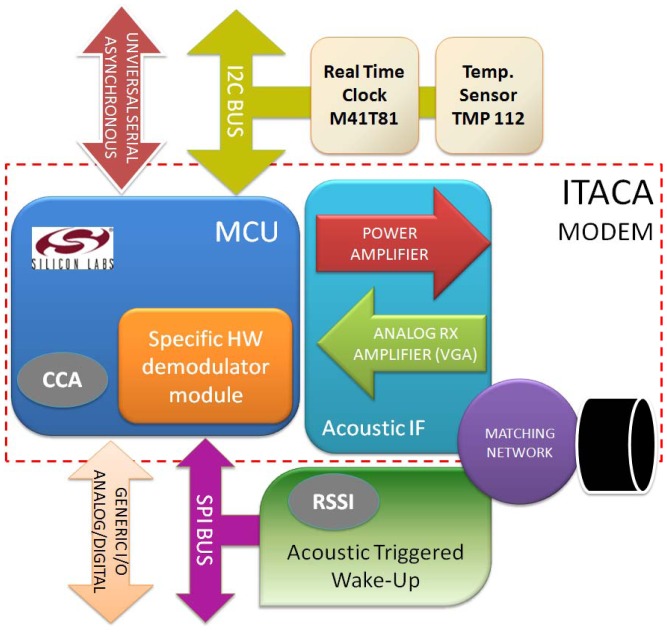
ITACA modem architecture.

The first description of the ITACA modem was given in [13]. However, the design has been improved with enhanced features such as AAW-U, CCA, and RSSI measurement. ITACA architecture is built around a microcontroller (MCU) and several integrated peripherals that only consume 12 mW in normal operation and less than 3 μW in stand-by mode. Figure 1 shows the overall ITACA block diagram architecture.

The first challenge was to adapt a transducer that does not increase the final price but enables sufficient distance and baud rate to be achieved. We decided to use a commercially available transducer that was significantly cheaper than hydrophones. Budget transducers are available for fish-finders from $50. In this way, the modem incorporates a transducer in the underwater link (matching network and transducer in Figure 1).

Secondly, input and output signals must be adapted to transducer-suitable frequency bands. The main micro-controller unit, with some hardware enhancements, implements a frequency-shift keying (FSK) modulation-demodulation using a coherent scheme.

Thirdly, acoustic waves are absorbed in water and so some type of amplification is necessary for transmission and reception. Transducer excitation for emitting an acoustic wave must be as efficient as possible. Moreover, before performing any demodulation algorithm, the electrical signal from the vibration of the piezoelectric transducer should be processed to fulfill two critical objectives: amplify the low incoming signal and eliminate as much noise as possible.

Fourthly, energy efficient enhancement is achieved when the physical modem layer is co-designed for the MAC layer optimal requirements. To save energy, the MCU should remain in sleep mode as much as possible. Waking the MCU can be achieved synchronously or asynchronously. Synchronous waking requires a clock source. As underwater temperature changes may affect low precision oscillators, high precision real-time-clocks (RTC) that can adequately support the expected temperatures and pressures are used. Moreover, a high precision RTC can be very useful in the configuration of TDMA-medium access. However, the objective of the ITACA modem is to be a flexible platform for the development of energy-efficient communication polices. Therefore, it also incorporates an AAW-U circuit. This wake-up system requires additional receiving hardware that must handle energy constraints without adding too much consumption to MCU requirements in sleep mode.

The same AAW-U module carries out carrier sensing and RSSI. The CCA software is implemented as an MCU routine (Figure 1). This low-cost, ultra-low power, and flexible modem can be used in underwater wireless sensor networks where a node consists of a modem with attached sensors. The following subsections analyze and describe the different blocks of the presented architecture.

### 3.1. Piezoelectric Transducer

**Figure 2.**
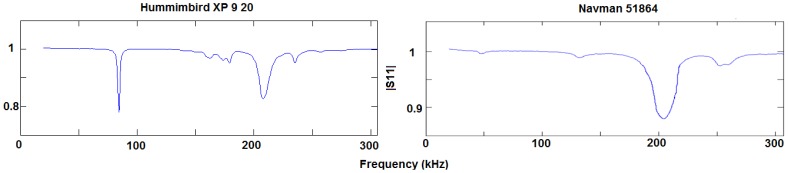
Frequency response of piezoelectric transducers: Humminbird XP 9 20 and Navman 51864.

The ITACA modem has been tested with two transducers: a Humminbird XP 9 20 [31] and a Navman 51864 [32]. Figure 2 shows the measure process results of the reflection coefficient according to frequency. Using a vector network analyzer (VNA) instrument, it was possible to determine incident and reflected wave ratios for both magnitude and phase. Both graphs in Figure 2 show on the vertical axis the reflection coefficient magnitude (also known as S11) across the frequency range in kHz on the horizontal axis. In those frequency ranges where the S11 magnitude is close to 1, it is expected that almost all the energy incident to the piezoelectric element will be reflected and so no acoustic wave is generated. Frequency bands in which this magnitude is closer to 0 are more suitable as communication frequency bands.

The the left-hand graphic shows that there are two identified bands around 85 kHz and 200 kHz (HUMMINBIRD device) that fit with the communication bands described. However, due to water attenuation, it is more worthwhile using the lowest possible frequency band: 85 kHz. In any event, the 200 kHz band is an alternative with more bandwidth and a more effective bit rate, and this should be taken into account for future designs that require a faster baud rate than 1 kbps. Some 300 ohms of pure resistive input impedance was calculated in the resonant frequency.

**Figure 3.**
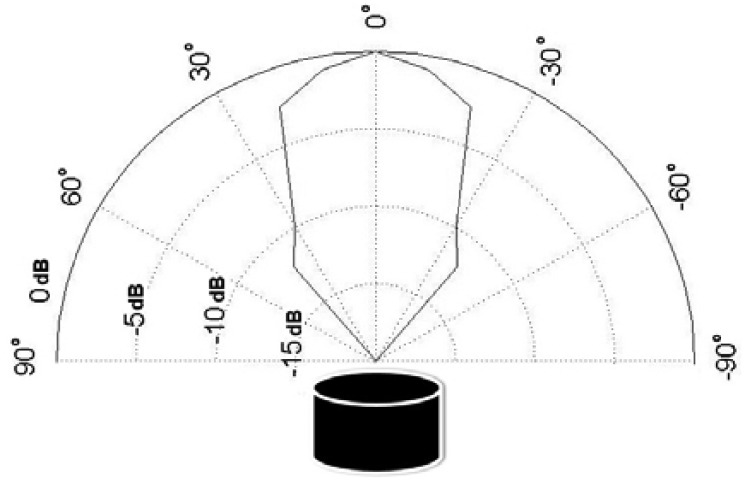
Measured HUMMINBIRD XP 9 20 beam geometry.

The Humminbird transducer has been characterized by measuring relevant parameters in this frequency band: transmitting sensitivity is 115 dB (re 1 μPa/V @ 1 m); receiving sensitivity is −154 dB (re 1 V/μPa @ 1 m) and the Humminbird transducer beam geometry is shown in Figure 3.

### 3.2. Modulation

ITACA reached 1 kbps with 1 kHz bandwidth (84.5 kHz frequency is used for symbol ‘0’ and 85.5 kHz for symbol ‘1’), which is clearly higher than other FSK solutions such as [17,21,22] or [33]. Moreover, 1 kHz bandwidth is not far from more complex modulation techniques, such as [34] (QPSK, 550 bps), [19] (PSK, 300–5 kbps), although ITACA has a lower power consumption than rModem and WHOI. Finally, there are high consumption modems that reach 16 kbps [18] or 75 kbps [35] and are very suitable for image transmission. Comparisons are shown in Table 1.

Coherent binary FSK *modulation* algorithms are very simple. The frequency of the transmitted signal changes according to the bit to be transmitted. This task can be performed using an internal peripheral counter and algorithm execution time is negligible.

**Figure 4.**
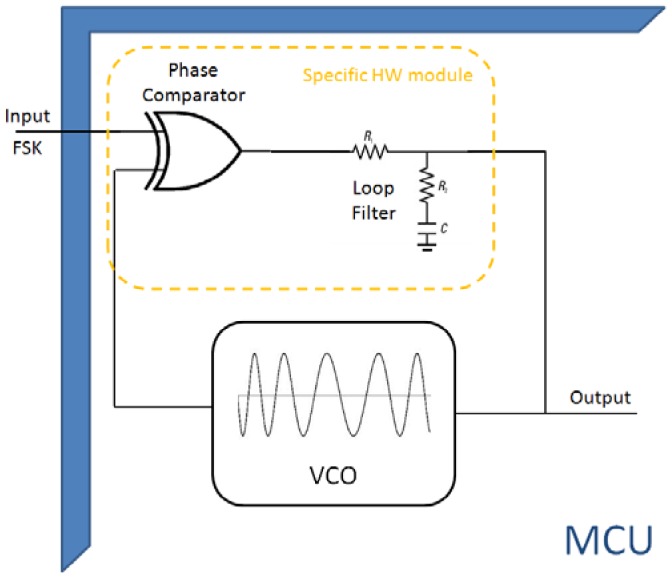
PLL block diagram.

However, *demodulation* algorithms are more complicated because phase-locked-loop (PLL) is needed. Figure 4 shows the proposed block diagram demodulation system. It is formed of three main blocks: a phase detector; a low pass filter; and a controlled oscillator. When the loop is locked, the ‘output’ net provides the demodulated signal. The phase detector is based on an XOR gate and filtering in a lead-lag low-pass filter. It is an energy-efficient HW-SW solution because both the external phase detector and the low pass filter relieve the microcontroller from the most consuming tasks in demodulation. Because no multiply-accumulate unit is needed, ultra-low power microcontrollers can be used. For example, the inexpensive c8051f920 Silabs microcontroller [36] is an 8-bit-microcontroller that offers very low power consumption.

### 3.3. Power Amplifier

Piezoelectric transducer excitation has been previously carried out mainly using three power amplifier types: class-B [34]; class-D [19,21,33]; and class-AB [37]. Although the class-B amplifier architecture uses just one power transistor, it is clearly inefficient because only positive voltages are used (50% of the input signal) and this leads to low efficiency. The use of commercial class-AB amplifiers can avoid the drawbacks of class-B amplifiers, but they suffer large power losses due to the simultaneous interaction of both transistors. Class-D amplifiers decrease the overall cost and increase power efficiency [19,21,33]; however, there are some drawbacks, such as the need for a complex impedance-matching net, the presence of greater electro-magnetic-interference (EMI), and a maximum frequency that is limited by the amplifier itself. Moreover, commercial class-AB and class-D amplifiers were originally designed for audio applications (low impedance and frequencies below 20 kHz), which is clearly out of specification for echo sounder transducers in both frequencies and impedances (300 Ω @ 85 kHz).

Consequently, we have decided to design a custom HW-SW power amplifier stage that maximizes the power that is transferred to the load and the efficiency. The objective is to avoid a simultaneous activation of both output push-pull stage transistors (Q1 and Q2) during transitions. If Q1 is completely shut down before Q2 becomes active and vice-versa, power losses will decrease and efficiency will be increased.

**Figure 5.**
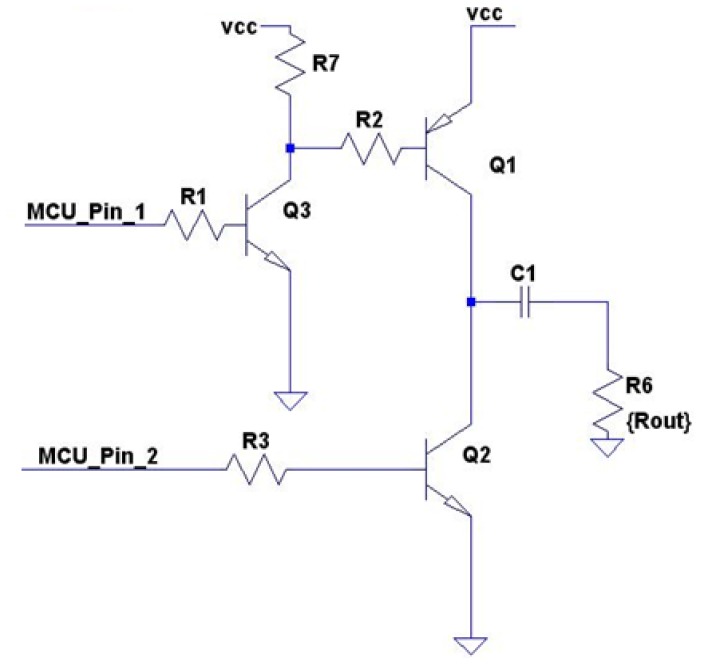
Digitally controlled push-pull B-class amplifier stage.

For that purpose, the amplifier shown in Figure 5 sets push-pull transistors that are independently activated or de-activated using two output pins on the MCU. The solution has been labeled as ‘push-pull class-B-based digitally controlled amplifier’ (D-PP-B). This architecture is especially suitable because harmonic distortion is not critical in frequency shift modulation applications.

The transducer sensitivity values shown above reflect the relation between the voltage applied to the piezoelectric element and the acoustic wave pressure produced. The higher the applied voltage, the louder the acoustic wave that is produced and the longer the distances that it can travel. Figure 5 shows that the maximum peak-to-peak voltage (Vpp) that can be applied to the transducer may be changed by modifying DC supply voltage (Vcc). This feature can be tackled using variable output power management devices.

**Figure 6.**
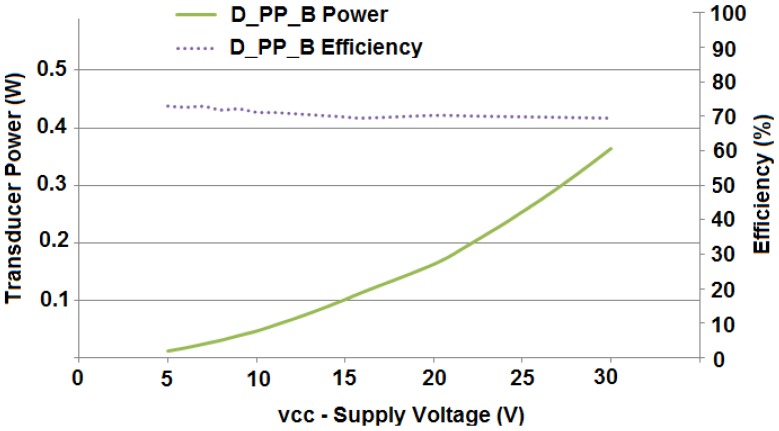
Amplifier output power and efficiency *versus* supply voltage.

Since the acoustic transducer has an input impedance value, if the applied voltage increases then its power increases quadratically, and the power dissipated by the amplifier also increases. Amplifier efficiency is defined as the ratio between supplied power and transducer dissipated power at a certain supplied voltage (Vcc). In an ideal scenario efficiency is calculated as one, as piezoelectric Vpp and supply voltage (Vcc) are equal. Power amplifier experiments are shown in Figure 6. Vcc voltages of +5 V, +10 V, and +15 V supplied to the amplifier stage dissipate 12 mW, 48 mW, and 108 mW, respectively. Measured efficiency is over 70%. As shown in the results section, we have reached 200 m with the highest voltage configuration (+15 V) and 20 m have been reached with +5 V.

### 3.4. Analog Reception Amplifier

**Figure 7.**
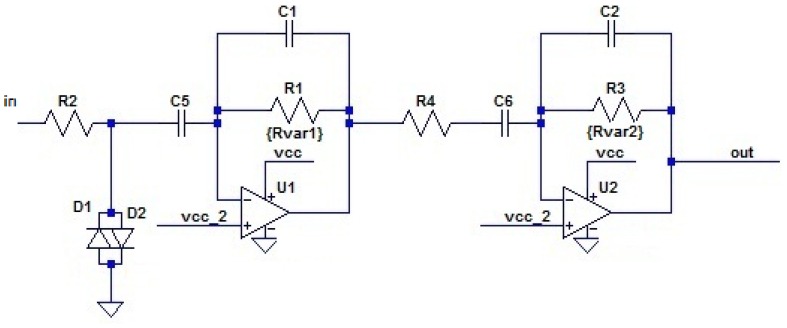
Analog processing stage schematic.

Figure 7 shows the ‘analog processing’ schematic implemented in the receiver. Instead of a simple pre-amplifier [38], the analog reception amplifier has been upgraded and includes a pass-band filter. Any interfering signal or noise in the communication bandwidth is therefore removed. This filter attenuates signals from the piezoelectric in the frequency band around 200 kHz (frequency range observed in the piezoelectric characterization).

The design introduces operational amplifiers that work correctly with signals around 85 kHz. Moreover, resistors labeled as R1 and R3 in the schematic of Figure 7 are digital potentiometers. The main microcontroller can vary the resistance of each of these resistors independently to modify the overall reception filter gain. This feature enables the implementation of an automatic gain control (AGC) that is very valuable in this type of system. Frequency response does not change significantly through all the resistor values; however, gain gradually changes from approximately 0 dB (no gain at all when the emitter is very close to the node) to 80 dB (maximum value).

In summary, the received signal is filtered and amplified by a two-stage pass-band filter based on single-supply operational amplifiers. An attenuation slope of 40 dB/decade can be easily achieved in the stop-bands by using two stages. Moreover, overall gain can be obtained by multiplying each individual stage gain. As operational amplifier maximum gain is inversely proportional to the goal bandwidth, gain equalization through different stages leads to an optimal ratio between the filter bandwidth and the maximum achievable gain, which in this case is 40 dB (×100) per stage or 80 dB (×100,000) overall gain.

To filter as much noise as possible before signal amplification, an additional three-stage L-C band pass passive filter has been inserted next to the piezoelectric element input. This network has been especially designed to match the transducer and the modem impedances. As a result, the final filter has a more selective band and attenuation slope of 80 dB/decade.

### 3.5. Wake-Up Systems

#### 3.5.1. Synchronous Wake-Up

To implement a synchronous WU, the modem includes a real time clock (RTC) with a 32.768 KHz crystal and a set of memory and configuration registers that store date and time [39]. Typically, an RTC can be programmed by the microcontroller via a serial interface. It also has one or more digital outputs that are activated when any alarm conditions are met. These outputs can be used as external inputs to the microcontroller and can wake the microcontroller from its deeper low-power modes. The RTC specifications are 2.7 V–5.5 V and 0.4 μA in stand-by mode; or 2 mA in fully operational mode.

Synchronous WU is widely used in the literature and its implementation does not imply any real innovation; however, this module remains very useful and has been included in the modem to enable the implementation of upper layer protocols.

#### 3.5.2. Asynchronous Wake-Up

In contrast, asynchronous acoustic wake-up (AAW-U) design is a considerable challenge in UWSN development since very little work has been done in this area. Previous work in this area with terrestrial nodes [40] showed that no extra hardware is needed to generate and transmit wake-up signals, while additional hardware is required to receive and analyze incoming signals and wake the MCU when needed. Although the use of this kind of system was theoretically analyzed in [25], in this paper the AAW-U system proposed is tested with a set of experiments in several scenarios.

AAW-U is carried out by an ITACA modem using as a core an off-the-shelf commercial peripheral designed for RFID tags: an AS3933 from Austria Microsystems [41]. Among its configurable parameters, it can work in the 65–95 kHz band, with simple carrier detection or carrier detection plus 16–32 bit pattern recognition. The operating supply voltage is 3.3 V—the same as the Silabs C8051F920. This wake-up peripheral has an ultra-low power consumption of only 2.7 μA.

The circuit has been completed with a specially designed matching net formed by a 3-stage T-structure band-pass filter with passive inductors and capacitors. This circuit was originally designed to be triggered using magnetic coupled signals with a coil antenna for RFID systems. Therefore, a net must be specially designed that matches both impedances and suitably couples the acoustic incoming signal to the RFID based WU circuit. We have found that this T-structure with inductors and capacitors is the most suitable structure given that capacitors avoid any circuit bias modification, and inductors mimic coil-antenna magnetic coupling.

##### WU Transmitter

When a modem needs to establish communication with another modem, it starts by sending a modulated on-off keying (OOK) WU signal. RF researchers generally use on-off-keying (OOK) signal modulation in the wake-up operation in order to save energy [26–28]. Moreover, OOK transmission can be made compatible with FSK (without additional hardware) by switching modem power amplifier output on-off according to the transmitted symbol (1–0). Bit synchronism is achieved by Manchester coding. It is obvious that OOK underwater transmission is very limited, but our system uses this transmission only for the WU signal, and not for payload or protocol control messages.

##### WU Receiver

The WU signal can be configured to be just ‘carrier frequency’ or use 8-bit or 16-bit patterns. Receiver power consumption is 10 μW. The WU pattern is pre-programmed into the modem as an 8 or 16-bit array. Thus, the modem will only respond to this programmed WU pattern.

**Figure 8.**
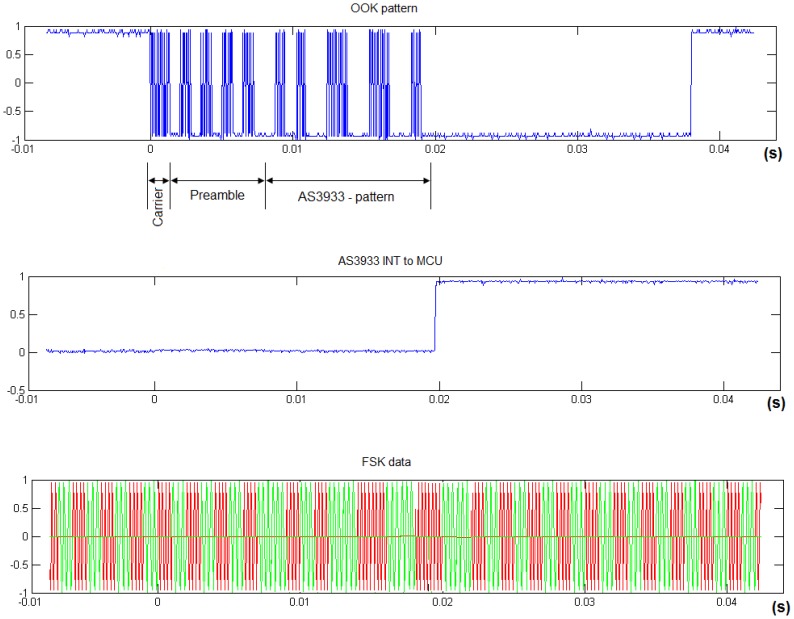
OOK detected pattern *versus* FSK data example.

Figure 8 illustrates the modem architecture embedding an AAW-U circuit. The analog reception stage has been designed to both filter and amplify incoming FSK signals; as well detecting incoming WU signals. WU signals and FSK data are sent on the same channel frequency range (which depends on the transducer). FSK incoming signals are adapted through the circuit in Figure 7; while WU incoming signals are decoded by the WU circuit that is directly connected to the transducer matching net.

Figure 8 shows that when the WU circuit is configured to recognize a pattern, the WU signal must include three elements: a carrier, a preamble, and a Manchester coded 8 or 16-bit array. These elements take 1ms, 7 ms and 12 to 16 ms respectively. Detection is performed by both an envelope-detector and a correlator embedded in the wake-up core IC. During the whole detection process, the MCU remains asleep, and the VGA is inactive at minimum possible power consumption. Eventually, following a positive WU signal recognition the IC will cause an external interrupt in the MCU. The interrupt handler manages MCU mode operation change; and no additional transducer is needed with this solution.

Selective wake-up configuration is validated in Section 4. False alarms can appear when FSK transmissions mimic OOK WU signals. When programming carrier frequency detection without pattern recognition, the WU signal only requires the carrier field shown in Figure 8 (1 ms). However, without pattern recognition, any emitted signal will wake up the modem given that the FSK and OOK carrier frequencies are the same. Therefore, this solution is only useful for special broadcast configurations.

Moreover, tests have shown that FSK data transmissions do not produce positive recognitions. Experiments were carried out in a 2.40 × 0.6 × 0.6 meter water tank with two modems: receiver and transmitter. The receiver was sleeping while the transmitter continuously sent random FSK modulated data. During a 24-h test, no MCU interrupts were reported. The reason is because FSK modulated signals are emitted with the same amplitude along with the packet. Therefore, the ‘1’ symbol is constantly recognized by the envelope detector as being non-coincident with the fields in Figure 8.

The system can be in two possible states. In the first state there is an OOK WU signal which does not produce effective information through the FSK demodulator. This signal can be decoded by the wake-up circuit which interrupts the MCU, and the interrupt handler should recognize the event. Alternatively, in the second state there is an FSK data signal that will be unrecognizable by the WU system and therefore, no wake-up interrupts will be set by the MCU during FSK data reception.

### 3.6. Clear Channel Assessment

CCA is a mechanism defined in standard IEEE 802.15.4 at physical level that enables implementation of CSMA based MAC protocols. Although this standard is not specified for acoustic underwater transmission, mechanisms such as CCA can be adapted to provide more flexibility to the MAC layer implementation. CCA can reduce collisions by listening to the channel before starting transmission.

IEEE 802.15.4 exploits three clear channel assessment (CCA) methods that are based on: detection of in-channel energy above a given threshold (referred to as mode 1); detection of an IEEE 802.15.4 compliant signal (referred to as mode 2); and a combination of modes 1 and 2 (referred to as mode 3) [30].

The CCA feature has been implemented as a physical-layer mechanism and an MCU software routine. Physically, CCA requires carrier frequency (CF) detection or RSSI observation, both of which are supported by the already implemented WU circuit without additional hardware.

The WU peripheral integrates a frequency detector based on a zero crossing counter. This counter counts the zero crossings of the input signal within a pre-defined time window. Additionally, the counted value presents some configurable tolerance. Our experiments have shown that with a suitable configuration, the peripheral detects incoming signals from 60–100 kHz. If CF is detected in this range, the peripheral enables an internal automatic gain control amplifier. Because 85 kHz is within the detectable frequency range, the solution is viable for detecting if the channel is occupied by either an FSK or OOK transmission. After CF detection, the gain of the amplifier is set to the maximum and the AGC reduces this level according to the received signal input level. After 1.06 ms, the AGC algorithm is complete and a stable RSSI value can be read from the corresponding register. The CCA implemented routine reads the RSSI value for eight symbols to obtain an average value above 2 dB.

**Figure 9.**
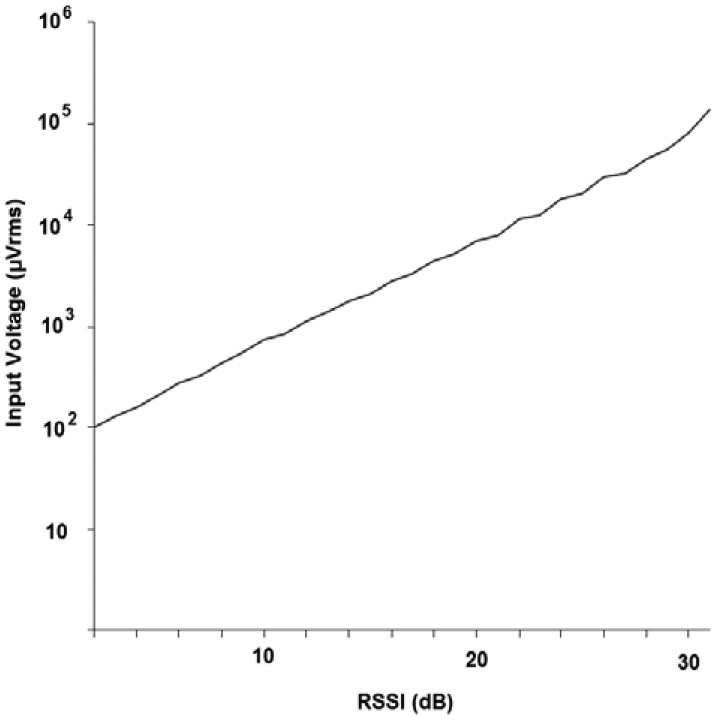
RSSI read for different input voltage signals.

Because the frequency range is wider than the modem transmission frequency, the CCA mechanism reads the RSSI value to obtain a more precise detection. The RSSI value is read by an MCU software algorithm executed before transmission. Figure 9 shows that the minimum RSSI read value is 2 dB.

Figure 9 shows the relationship between RSSI registered values and signal μVrms. Signal strength is measurable above 100 μVrms, which corresponds to the wake-up module sensitivity. This level can be improved to 80 μVrms, thereby enabling an internal 3 dB additional amplifier.

## 4. Experimental Analysis

### 4.1. Energy Efficiency

The highest measured power consumption in full operational mode was 24 mW, and this represents the lowest consumption published until now. This figure is for both the analog reception amplifier and the MCU consumption. In [21] the reported value is similar (25 mW), but the RX consumption reported in previous works was generally higher than 200 mW [19–22,33–35].

**Figure 10.**
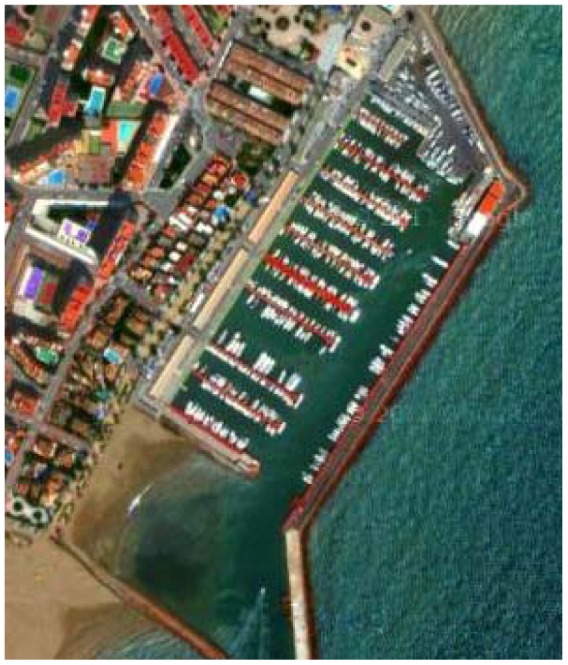
Aerial view of the small marina experimental scenario.

The transmission distance reached depends on transmission power amplification. The ITACA modem has been tested with 12 mW, 48 mW, and 108 mW. In October 2011, the modem reached 240 meters with 108 mW in a small marina shown in Figure 10. This short/medium range is useful for the off-shore and coastal monitoring applications that are currently funding the modem development. Overall modem consumption in transmission will be 108 mW + 12 mW (MCU consumption). This is the lowest reported value for short range links.

When no data is transmitted, the modem remains in sleep mode awaiting a wake-up event. Consumption is reduced to less than 3 μW from the stand-by MCU mode, and 8.1 μW from the WU peripheral. This is the lowest consumption reported until now. It has been demonstrated that this solution represents a 97% energy savings when compared with the state-of-the-art modem with asynchronous wake-up capability [25].

### 4.2. AAW-U Reliability

Underwater applications are diverse and range from the single communication of a pair of modems to dense networks. Wake-ups can be caused by a single carrier frequency detection or additional pattern recognition. Both possibilities are configurable in the WU system and with equal consumption levels. Although single carrier frequency detection can be useful for peer-to-peer or broadcast communication, pattern recognition is very desirable in clustering or tree topologies with more than two nodes per receiver. For example, when a submerged node, formed by the modem and several attached sensors, wants to transfer data, it will send a selective wake-up only to its target—thereby not waking neighboring nodes.

To evaluate system reliability, two experiments were made to measure false wake-up events caused by noise or neighboring transmissions. Firstly, we wanted to determine if false wake-ups can occur within the simplest configuration of single carrier frequency detection when subjected to real environmental noise. Experimentally, the modem remained submerged 24 h in a marina (Figure 10). There was no registered wake-up. Moreover, this experiment is extensible to pattern recognition because in addition to single carrier frequency detection, the probability that noise could compose a valid preamble plus a Manchester coded pattern is practically negligible.

Secondly, having discarded false wake-ups due to environmental noise in a real scenario, false wake-ups were measured in a controlled scenario when subjected to OOK pattern transmissions. In this experiment, two modems were submerged during 24 h in a water tank. One of these modems was constantly sending WU signals with 16 different 8-bit array valid patterns. The receiver modem should only recognize one of these patterns.

Two cases have been evaluated with this experiment. Firstly, the number of false wake-ups was evaluated, which is the number of MCU interruptions that occur when the programmed pattern was not transmitted. These errors lead the modem to become active when it was not required and, consequently, waste power. Secondly, the number of non-detected transmissions of the programmed WU pattern was evaluated. This condition can lead to communication failure as the microcontroller is not correctly woken.

**Figure 11.**
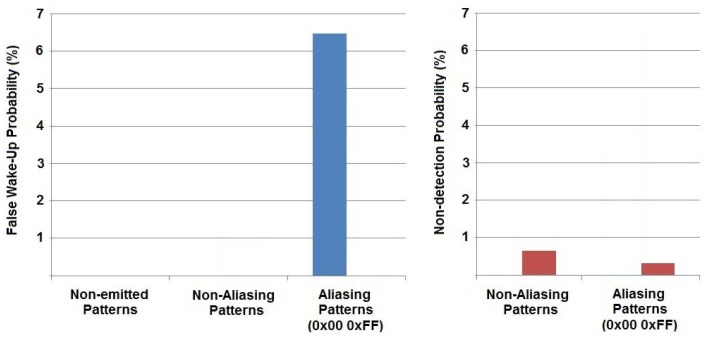
Results obtained emitting different patterns (0x00, 0x7F, 0x55, 0X0F, 0X01, 0X21, 0X51, 0XFF, 0X80, 0XAA, 0XF0, 0X81, 0X88, 0X92). Non-emitted patterns (**left**): reception pattern configuration different from emitted patterns. Detection pattern errors (**right**): no detection of actually emitted patterns. Aliasing evaluated with all eight patterns.

Two scenarios were set. In the first experiment, the WU pattern is never sent. This scenario characterizes false waking probability. In the second scenario, the detectable WU pattern is transmitted periodically. Results are shown in Figure 11.

The wake-up circuit topology itself can lead to some pattern aliasing: more than one pattern can cause positive pattern detection when only one was configured. The inner flexibility of the correlator causes this aliasing effect for those patterns whose Manchester encoding is confusing when compared to the preamble structure. For example, the worst case has been measured for 0xff pattern and these results are included in Figure 11 in the ‘Aliasing’ column. An 0xff pattern, combined with the preamble (see Figure 8), can be easily confused by the correlator with an 0x00 pattern and *vice-versa*. In fact, 0x00 patterns caused a false wake-up when the 0xff pattern was configured. Since 16 different patterns were emitted and 0x00 was always false detected, it means that Figure 10 shows around 6% false wake-up detections.

If all these aliasing patterns are avoided, the performance of the WU system increases. The worst case observed with non-aliasing was 0x88-bit array. No false wake-ups were reported but, in this case, non-detections occurred at a rate of 0.6% (Figure 11 non-aliasing column).

The non-emitted column shows the results when the configured pattern in the receiver is not emitted. Neither false wake-ups nor non-detected pattern were reported. Of course, this condition is extrapolated for those patterns with no aliasing effect. Finally, pattern recognition can be reinforced by switching the acoustic interface to FSK communication. However, the advantage of our solution is low consumption with selective wake-up support.

### 4.3. CCA Mechanism Evaluation

Similar to IEEE 802.15.4 CCA mode 3, ITACA modem combines carrier sense with an energy above threshold. RSSI signal level measured by the AAW-U HW module is read during 8 bit cycle time (8 ms)—8 symbols to FSK modulation—using an SW routine executed by the MCU. Channel is assessed as clear if all the RSSI read values are below a predefined threshold level (10 dB).

The reliability of the proposed solution has been tested in a 24-h experiment. The experiment consisted of monitoring the activity of two modems. One modem emitted a FSK modulated signal during 50 s and released the channel during 50 s in an endless loop. On the other side, a second modem tried to emit information every 5 s. In case of a positive CCA (clear channel), the modem transmitted a packet and reported the transmission. Otherwise, the modem did not transmit a packet and reported a non-possible transmission. Table 2 shows reported data.

Test results differentiated two cases: Node 1 silence and Node 1 transmissions. During Node 1 silence, Node 2 transmitted packets during every attempt. However, when Node 1 was occupying the channel with a transmission, Node 2 reported only a 0.7% rate of channel-clear-assessment errors. The proposed mechanism therefore presented a reliability ratio of 99.3%.

There is one restriction to this solution and this relies on the FSK modem sensitivity (30 μV). Minimum wake-up peripheral sensitivity is 80 μV. Consequently, there is a gap in the incoming signal voltage between 30 μV and 80 μV in which the CCA algorithm could report a clear channel and a collision could occur. This collision scenario depends on modem distance and transmission power. For example, for a 108 mW transmission power and a distance under 240 meters, incoming signal strength would still be above 80 μV. However, signal strength beyond 240 meters decreases below 80 μV and the CCA algorithm will be affected by this sensitivity gap. However, this solution is very suitable for short/medium distances because no new hardware, nor RSSI estimation algorithms, nor extra power consumption is needed to implement the mechanism. In summary, the modem architectural design has been successfully tested and will be used to develop UWSN for off-shore monitoring, seabed to surface coastal links, and mooring applications.

## 5. Conclusions

This paper presents an ultra-low power and low-cost acoustic modem that enables the deployment of maintenance-free, long-life submerged nodes. The ITACA modem architecture combines a typical microcontroller-based core with energy-efficient mechanisms. One of the most power hungry elements of an acoustic modem is the processing requirement in reception phase (demodulation). The proposed solution uses coherent-FSK modulation and a combination of hardware and software that requires only 24 mW for receiving data. Moreover, with 108 mW of transmission power, the modem has been tested to transmit data at 240 m—which implies an overall consumption of only 120 mW. Although the modem achieves a mere 1 kbps data rate with a carrier of 85 kHz—forced by the piezoelectric transducer used in the current implementation, this relatively low transmission speed is sufficient for sensor pay-loads containing temperature, pressure, and conductivity measurements.

A key novelty is the adaptation of RFID technologies to acoustic transmission. This idea has facilitated the design of an asynchronous wake-up mechanism that consumes only 8.1 μW and enables the recognition of 8 or 16-bit patterns without MCU activation. This asynchronous wake-up design has been tested for non-detections and false positives with excellent results. The distance covered by the WU with OOK modulation was also 240 m. Finally, the system incorporates clear channel assessment (CCA) at both carrier frequency detection and RSSI level. The CCA mechanism has been tested experimentally and provides a reliability of 99.7%.

## Figures and Tables

**Table 1.** Modulation and data rate comparison.

	**[34] rModem**	**[19] WHOI**	**[33] AquaModem**	**[21] USC**	**[17] UCSB**	**[33] AquaNode**	**[22] NCSU**	**ITACA**
Modulation	QPSK	FHFSK/PSK	MFSK	FSK	FSK	FSK	FSK	FSK
Data rate (bps)	550	80/5,000	133	600	80	48	31	1,000

**Table 2.** CCA experimental data.

Node_2 messages sent	7,978
Node _2 attempts	15,653
CCA failures	111 (0.7%)
